# False Impressions

**Published:** 1888-10-13

**Authors:** Caroline Fothergill

**Affiliations:** Author of "An Enthusiast," "A Voice in the Wilderness," etc.


*(To be continued.)*

### FALSE IMPRESSIONS.

By CAROLINE FOTHERGILL, Author of "An Enthusiast," "A Voice in the Wilderness," etc.

*(Continued from p. 14.)*

### CHAPTER II.—IN THE WAITING ROOM.

A LADY and gentleman were seated in Dr. Temple's waiting room. They had arrived almost at the same moment, and within a quarter of an hour of the time when the doctor was supposed to dismiss his last patient. Immediately after their arrival, a lady and gentleman, evidently husband and wife, had been summoned to the consulting room and they were still there half-an-hour later. All this time these two had sat in perfect silence. They were strangers, and apparently not sufficiently interested in one another to speak, yet there was an air of distinction about both which rendered them noticeable. The lady was young and beautiful, perhaps four and twenty years of age, with a tall, finely moulded figure, and a face at once sweet and imperious, with delicately chiselled features and a determined expression. Her eyes and hair were brown, and her complexion clear and pale, a pallor which, however, took nothing from her appearance of perfect health; and her companion as he looked at her again and again was, more puzzled each time to account for her visit to the fashionable physician of the fashionable watering-place of Clearwater. Her name was Beatrice Stanford, and some particulars which her companion did not know until long afterwards, may as well be given here at once. She was rich and her own mistress. Her father had been a retired Indian officer, who had inherited the family property rather late in life, and who had inherited a large fortune from his mother. His wife, too, had been rich, and all the accumulated wealth of both parents now belonged to their only child. She had never known her mother, and her father had died soon after her twenty-first birthday. After his death she had lost no time in inviting her old and only intimate friend, Mrs. Ledward, who had recently lost her husband, to share her home, her life, she said, but Mrs. Ledward had laughed at her, saying she should never look upon herself as other than a tenant-at-will. Mrs. Ledward was some years Beatrice's senior, and a very charming woman; a most desirable companion in every way, except that she had very little influence over Beatrice, and what influence she had was of affection rather than character. That which Beatrice wished to do, she did, undeterred by Mrs. Ledward's approval or non-approval. Mrs. Ledward was fully aware that her influence over her friend was very limited; she confessed it with charming candour, but then, she added, in extenuation, no woman ever would have much influence over Beatrice, she would bow to one person alone, and that person would be her husband, if she ever married Neither was Miss Stanford an easy person to live with. She was communicative or reticent as she felt inclined at the moment. She was imperious and quick tempered, and impulsive, and perhaps no woman but Laura could have lived in harmony with her. But Laura had known her since her babyhood, she knew Beatrice's faults, and how they had been encouraged all her life. she knew, too, that Beatrice was keenly alive to them, and had hard fights to overcome them. So although strangers and acquaintances were apt to say of Miss Stanford that she appeared to think the world had been created for her sole convenience, Laura knew the generous nature which lay under the surface, and her love was deep and strong. Beatrice was just now—for a purpose which will be explained immediately—seated in Dr. Temple's waiting-room, with nothing to do but observe her companion. She saw a tall man, thin, but wiry, with strong features, and dark complexion. Even watching him as he sat glancing over the newspaper which he had taken up from the table, she found that his face was a changeable one, that sometimes he looked positively plain, and again just missed being handsome. She did not exactly admire his face, and yet she found something attractive in it; something which compelled her to look at it again and again, and wonder what manner of man he was to whom it belonged. He looked cool and rather cynical—the very reverse of her own disposition. She both liked and disliked the face, and she could not tell which feeling predominated.

Before she had finished her scrutiny, she was called to the consulting-room, and her companion was left alone. The opportunity may be taken of telling the reader who he was.

He bore the name of George Murray, and in some respects his circumstances were not unlike those of Beatrice Stanford. Like her, he was rich and free. He had been the only son of his parents, who had both died many years before this. He did not live at Clearwater, but at the large provincial town of Mayfield, at the other side of England, and his time was more fully occupied than that of many an ever-busy man who works for his living. As a boy, he had been grave and thoughtful beyond his years, and as he became a man this disposition grew with him. He was interested in many things, his keen hunger after new interests, and the zeal with which he pursued them was one of his most marked characteristics. His special aims will be gone into in the course of this story, so it is needless to enter into them here.

He lived with his widowed sister, a woman fifteen years older than himself; he was six and thirty. There was not much sympathy between them, but she had offered, on her husband's death, to come and keep house for him. He had agreed to a temporary arrangement, and she had stayed on, strengthening her position by rendering herself a necessary evil, until George had ceased to think of suggesting any change. She clung to her home with him, because he gave her consequence and a position, and the skeleton in her cupboard was her fear lest George should marry. She discouraged female society, and never invited any woman younger than herself, or with grown-up daughters to her house. The consequence of this was that George, having no intention of marrying and being very indifferent to women, imagined his sister to be a very fair sample of her sex. He was not blind to her faults: so that his general idea about women was that they were both weak and violent; that they loved dress and hated to be contradicted; that they had very little reasoning power, and that they could, when they thought occasion rendered it necessary or advisable, lie, to gain their ends even while they professed a shuddering horror of falsehood in the abstract. He also believed that they were timid creatures, subject to needless alarms and unreasoning panics, and not to be safely trusted with the guidance of their own lives.

These were his present convictions. There might be other and different women, but he had not met them; and how far his convictions were to be modified this story will partly show.

A quarter of an hour went by before Dr. Temple dismissed Miss Stanford, and then he heard them come out of the next room. They went along the hall, the doctor speaking in his naturally suave and dulcet tones. What he said, George could not have heard, even had he listened; then came a woman's voice in reply, clear and distinct, so that every word reached his ear.

"I am much obliged to you. I regret very much having taken up your time with so small a matter."

Then the house door closed, and immediately afterwards the doctor joined him.

"Welcome," he said, holding out his hand. "Come into my den, and have a smoke."

George accompanied him to the den—for it may as well be stated at once that George Murray was not a patient of Dr. Temple's, but an intimate friend; that he was leaving Clearwater on the following day, and had come for a final chat.

"Who was that lady who has just left you?" asked George, plunging at once into what he wanted to know.

"I can't tell you," said his friend. "I never saw her before, and the chances are I shall never see her again. She does not live here."

"No more do I. But tell me something about her." "What do you know of her?"

"Nothing, but we sat together for more than half-an-hour, and she seemed, as far as I could judge, to be getting more and more out of temper with you every minute."

The doctor laughed. "Did you not speak to her?"

"Heaven forbid!" said George piously. "I tell you she was out of temper, and what man in his senses would address an unknown lady in that frame of mind. I judged from her expression that she would pour out the vials of her wrath upon you."

"She was very angry with me for keeping her waiting. I fancy she has not been used to it; but before she left, she was more angry with herself."

"What was the matter with her? To my unprofessional eyes she seemed in perfect health."

"She is; it was a purely imaginary disorder which she came to tell me of."

"Ah," said George, with a smile which was not purely benevolent, "a woman's maggot; I fancied as much."

"Ay," said the doctor, "you're an astute fellow, I've no doubt, and can see through a barn door as well as the rest of us."

"Well, tell me about her," persisted George. "I feel interested in her; tell me the whole case."

And Dr. Temple told it, as he often told things confidentially to his friend, George Murray, finishing with—

"And, as I said before, it was purely imaginary; her throat was absolutely free; there was not the shadow of an obstruction in it."

"Did you tell her so?"

"I told her so, frankly."

"How did she take it? There is nothing women hate so much as the truth."

'She took it very quietly, and said remarkably little; but from that moment her anger was shifted from me to herself. One can see she is proud, and I think she thought she had been very foolish."

George threw back his head and laughed heartily.

"You thought so, no doubt," he said.

"Well, no, I didn't. It was pure nervousness which had brought her, and at first sight it is difficult to reconcile nervousness like that, with a physique such as she has; still, the two do exist together—she was a striking instance of it.

"She seems to me very like most women, exceedingly nervous, and unable to reason her fears away."

"Well, you're wrong," said his friend. "In the presence of danger that woman would be as cool as you or I, probably cooler than you."

"Oh, no," said Murray, "that is nonsense."

"No nonsense at all. There are such women, and she is one of them. For an imaginary thing she runs for help in a hurry, and is angry with herself as soon as she finds out there is nothing wrong. But put that woman in a difficult or dangerous position, needing courage and endurance, and she would come out grandly."

Murray shook his head, but said nothing. What the doctor had just said was as a sermon in an unknown tongue. He scarcely believed in the existence of such women.

"You are romantic," he said at last, "and it is no use arguing with you, especially as I don't suppose we shall either of us ever see Miss Stanford again. Where does she live?"

"She did not say. She only told me, in answer to a question, that she did not live here, but was only passing through."

"Well," said Murray, "odd things happen, and if I ever meet her again I will let you know. You do the same for me. Whether you are right or not, I feel interested in her. She was a beautiful woman."

"A very beautiful woman," said the doctor emphatically.

"Did you give her any physic?" asked Murray after a long silence, and the doctor smiled to himself at this proof of what had been occupying his friend's thoughts.

"I wrote her a prescription. I daresay she is run down a little, or she would not have got such an idea into her head."

"Well," said Murray, "I should like to hear of her again sometime; but she is not a woman to my taste, anyway."

Meanwhile Miss Stanford walked away from Dr. Temple's house, holding between the tips of her fingers the envelope containing the prescription which he had given her. She went on until she came to a druggist's shop, which she entered, and, handing the prescription across the counter, commanded rather than asked that it should be made up and sent to her. The shop was close to the road in which she was staying, and she was at home in a few minutes.

She was greeted with some excitement by Mrs. Ledward, who plied her with many questions which she put aside until they had dined and were settled in the drawing-room with their coffee.

"I was really quite relieved to see you back. I could not imagine what had become of you. I began to think you must have been ordered off to the hospital," said the vivacious elder lady; and she laughed as she spoke. "But begin at the beginning. I suppose you found him in, and had to wait, since you were away so long."

"Yes, he was in," answered Beatrice, in her melodious and rather deep voice. "But just after I got there a lady and gentleman were summoned, and I suppose it was a serious case, for they stayed a very long time. I got so impatient, and felt so angry with myself for having gone at all. Several times I was on the point of coming away without waiting to see him."

"But what did you say when you did see him?" pressed her companion.

"I told him what you know—that I had been eating in haste, and a fish-bone had stuck fast in my throat, and refused to be dislodged."

"Yes," said Laura; "what did he do?"

"He put an instrument down my throat. It was very disagreeable, and he said he could see nothing. Then he asked questions, and I began to feel more annoyed than ever at having gone at all."

"Of course. After that?"

"Then he lit a lamp, and seated me before it, and himself in front of me. He put something else down my throat, and we sat there until I was almost suffocated."

"How nasty! But did he get the bone out for you?"

Beatrice coloured, and raised her head a little as she answered,

"He said there was no bone—there was nothing in my throat at all. It was all imagination and nervous fancy."

There was silence. Laura struggled hard to keep grave, but, after a fight with herself, she lay back in her chair, and burying her face in her handkerchief, laughed as if she were never going to stop.

Beatrice watched her for a moment, and then broke out almost passionately—

"I never felt so foolish in my life. Nothing but increasing discomfort for days would have induced me to go to a doctor at all. I have always disliked the profession; I have laughed at them, and refused to make their acquaintance. I have ridiculed women who were always in a state of rapture over their medical man, and I have likened the medical man to a pet dog—a necessary part of every lady's belongings. I have been proud of saying that I have no doctor; I have gloried in my perfect health, and now——"

She stopped short, and Laura sat up and dried her eyes, saying brokenly—

"I beg your pardon, Beatrice; it was rude to laugh, but I could not help it."

"Oh, laugh—laugh as much as you like. I could laugh myself if I did not feel such an idiot."

"It is just for that you ought to laugh. The first lesson taught to children should be to laugh at themselves. It is difficult to begin when one is a woman."

Beatrice said nothing. She was standing with one foot on the fender, looking half-amused and half-annoyed. Laura, after looking at her for a minute or two, went on severely—

"I know you think all these things, and it is very silly. You have no reason for thinking so. Just because you are never ill yourself you fancy no one else need ever be, and then, like most people who never have an ache or a pain, you go and get into a panic about nothing, and make yourself ridiculous."

Beatrice still said nothing. She really felt humiliated, and could not deny that there was much truth in what Laura said. Laura herself, somewhat softened by this mildness, went on more gently:

"Now tell me some more. What happened after he said it was all imagination?"

"Not much. He said he wanted me to take some medicine."

"I admire his courage. What did you say to that?"

"I said I had not taken medicine since I was a child, and I did not think it would agree with me."

"I admire your courage. How did he look then?"

"I don't know; he had his back to me."

"What a pity."

"You mean he was laughing at me. I don't care if he was. I wished him to know that I am not in the habit of poisoning my system with drugs."

"He might have retorted that nervous fancies poison the system just as much."

"The idea of me having nervous fancies is too ridiculous. I suppose he thought I was afraid there was something the matter with my throat, for he assured me more than once that the feeling was 'purely subjective.' It is most unlikely that anything should ever be the matter with me; and if he had any eyes—any professional eyes, I mean—he must have seen how strong I am."

"But about the medicine," said Laura; "are you going to take it?"

"Oh, yes," shrugging her shoulders, "I will take it. I left the prescription at Evans' on my way home."

Later in the evening Laura reverted to the subject, saying,

"By the way, were you alone all the time you were waiting?"

"No, there was a gentleman waiting too."

"Oh! who was he?"

"I don't know; I never saw him before."

"What was he like?"

"He was plain, but there was something distinguished about him," she answered; and then, after a moment's silence, she went on:

"I have made up my mind never to admit this visit. I can still say I have not been out of health since I was a child. I did not go to see Dr. Temple about my health; I am perfectly well. Let this be the beginning and end of the whole affair; we will never mention it again."

"I am at your orders," said Laura.

And having thus issued her orders, Beatrice dismissed from her mind both Dr. Temple and the gentleman she had left in the doctor's waiting-room, until, three months afterwards, she saw her "ghost" at Mrs. Lennox's garden party.

